# Verbena Attenuates Adriamycin-Induced Renal Tubular Injury via Inhibition of ROS-ERK1/2-NLRP3 Signal Pathway

**DOI:** 10.1155/2022/7760945

**Published:** 2022-09-28

**Authors:** Yicen Zhou, Qijing Wu, Zhenfang Du, Min Huang, Kun Gao, Xiaowei Ma, Hui Zhang, Sheng Qiang, Wei Sun

**Affiliations:** ^1^Department of Nephropathy, Center Laboratory, Zhangjiagang Traditional Chinese Medicine Hospital, Affiliated to Nanjing University of Chinese Medicine, Zhangjiagang 215600, Jiangsu, China; ^2^Department of Traditional Chinese Medicine, Huai'an First People's Hospital, Nanjing Medical University, Huai'an 223399, Jiangsu, China; ^3^Division of Nephrology, Affiliated Hospital of Nanjing University of Chinese Medicine, Nanjing 210004, Jiangsu, China

## Abstract

Chronic kidney disease (CKD) has become a global public health problem. Tubular epithelial cell injury plays a vital role in the progression and prognosis of CKD. Therapies to protect tubular cells is the key to delaying CKD progression. Our study found that verbena, a natural traditional Chinese herb, has a potential reno-protective role in kidney diseases. However, the detailed mechanism remains unknown. In the current study, we employed adriamycin (ADR)-induced renal tubular cell injury to mimic the conditions of tubular injury in vitro. Results showed that total aqueous exact of verbena (TAEV) ameliorated ADR-induced cell disruption, loss of cellular viability, and apoptosis via inhibition of ROS-ERK1/2-mediated activation of NLRP3 signal pathway, suggesting that TAEV serves as a promising renoprotective agent in delaying the progression of CKD, while ROS-ERK1/2-mediated NLRP3 signal pathway might be a novel target in treating kidney diseases.

## 1. Introduction

Chronic kidney disease (CKD) has become a global public health problem [1]. During the process, tubular epithelial cell injury was considered a direct cause of acute renal failure and the vital factor leading to irreversible progression of CKD [2]. Tubular epithelial cell injury destroys the tubulointerstitial structure, significantly enhances the migration and secretion of inflammatory cells and results in massive generation of chemokines, proinflammatory factors, profibrogenic factors, and matrix proteins, leading to the formation and development of renal interstitial fibrosis [3]. Hence, protection of tubular epithelial cells plays a vital role in delaying the progression of renal fibrosis and CKD. Exploring effective renal tubular protective medicines has become a common pursuit for nephrologists.

Verbena is a natural traditional Chinese herb widely used in hepatitis, gonorrhea, dysentery, nephritis edema, and so on [4]. Several studies reported the extensive biological activities of verbena, such as antitumor, analgesic, neuroprotective, immunoregulatory, and antioxidative properties [5–9]. Our clinical practice discovered that a prescription mainly composed of verbena significantly delayed the progression of renal function and reduced proteinuria in patients with diabetic nephropathy, indicating that verbena may play a promising role in renal protection. However, the concrete mechanism remains unclear. The study of the exact mechanism contributes to develop more novel therapeutics in renal diseases.

Adriamycin nephropathy (AN) is a classic pharmacological disease research model simulating the occurrence and development characteristics of tubular injury during the progression of CKD [10]. Accumulated evidence indicates that oxidative stress injury and inflammatory response underline the toxicity of ADR in tubular injury. Among them, activation of NLR family pyrin domain containing 3 (NLRP3) inflammasome and mitogen-activated protein kinases (MAPKs) have been demonstrated to be involved in the process [11]. Whether they participate in the protection of verbena against ADR-induced tubular injury remains unknown. Herein, our study aims to demonstrate the potential effects of verbena in ADR-elicited renal tubular injuries and explore possible mechanisms involved.

## 2. Materials and Methods

### 2.1. Reagents

Adriamycin (ADR) and N-acetylcysteine (NAC) was purchased from MCE (Shanghai, China). Verbenalin, hastatoside, and acteoside were obtained from Macklin (Shanghai, China).

### 2.2. Preparation of Total Aqueous Extracts of Verbena (TAEV)

Verbena (batch number: 210726010) was purchased from Suzhou Tianling Chinese Herbal Medicine Co. Ltd., and identified as authentic by the Chinese Pharmacist, Director Yu Hui of Zhangjiagang Hospital of traditional Chinese medicine. TAEV is a botanical extract of the parts above the verbena ground after drying. 0.5 kg of dried verbena was soaked in 2 L of water for 0.5 h and decocted for 1 h. After filtration, the aqueous decoction was made into a dry extraction powder under a vacuum concentration tank. The dried powder was then dissolved in ddH_2_O for experiments. The quality of TAEV was controlled using high performance liquid chromatograph analysis.

### 2.3. High Performance Liquid Chromatograph Analysis

Control standard samples including verbenalin (4.86 mg), hastatoside (4.13 mg), and acteoside (5.17 mg) were dissolved in 5 mL methanol. Take 1 ml each, add them together and quantify to 10 ml with methanol. TAEV (20 mg) was dissolved in 10 ml methanol. Concrete chromatographic conditions: chromatographic column: thermoC18 chromatographic column (250 mm × 4.6 mm, 5 *μ*m); detection wavelength: 238 nm; column temperature: 30°C; mobile phase: acetonitrile (A) −0.05% phosphoric acid aqueous solution (B); gradient elution: 0∼30 min, 14%∼40% A; flow rate: 1 ml/min, sample size 10 *μ*l.

### 2.4. Cells Culture

Normal rat proximal tubular epithelial cells (NRK-52E) were incubated in DMEM/F12 medium added penicillin G (100 U/mL), streptomycin (100 mg/mL), amphotericin B (0.25 mg/mL), and fetal bovine serum (5%) at 37°C with 5% CO_2_.

### 2.5. Animals

Eight-week-old male Sprague–Dawley rats (*n* = 24, 200–250 g) were obtained from the Animal Center of Yangzhou University (Yangzhou, China). After adaptive feeding for a week, rats were randomized into four groups: normal control (*n* = 6), adriamycin model (AN) (*n* = 6), TAEV (*n* = 6), and adriamycin + TAEV (AN + TAEV) (*n* = 6). This work was conducted in conformity with the guidelines for the Care and Use of Laboratory Animals of the Affiliated Hospital of Nanjing University of Chinese Medicine's Research Ethics Committee. The Research Ethics Committee of the Affiliated Hospital of Nanjing University of Chinese medicine authorized the protocol. The right nephrectomy was performed on the rats in the AN and AN + TAEV groups under isoflurane gas anesthesia. After surgery, each animal was injected with 200,000 units of penicillin every day for three days. On day 14, the AN and AN + TAEV groups received a tail vein injection of 4 mg/kg adriamycin. TAEV (2 g/kg) was administered through gavage once daily to rats in the TAEV group, whereas rats in the AN and TAEV groups received 2 mL of normal saline via gavage daily for two weeks. On day 21, the second adriamycin (2 mg/kg) injection was delivered. On day 28, every animal was slaughtered and blood, urine, and kidney samples were taken.

### 2.6. Assessment of Cell Viability

Cells were insulted with different stimuli as planned. After incubation, the medium was replaced with fresh one and added cell counting kit-8 (CCK-8) reagent for 45 min. Optical density (OD) was detected with a spectrometer at a wavelength of 450 nm.

### 2.7. Detection of ROS

Cells were exposed to various stimuli as plans and rinsed twice with PBS. Then, the oxidation-sensitive fluorescent probe (DCFH-DA) was added and incubated at 37°C for 30 min. Fluorescence images were captured using an inverted fluorescence microscope.

### 2.8. Assessment of Protein Oxidation

Protein lysates were prepared by sodium dodecyl sulfate (SDS) lysis buffer mixed with protease inhibitor cocktail (Thermo Fisher Scientific) and 50 mM DL-dithiothreitol. 5 *μ*L of 12% SDS and 10 *μ*L of 2,4-dinitrophenylhydrazine solution were added to each test tube (5 *μ*L). After incubation for 15 min at room temperature, each tube was then added 7.5 *μ*L of neutralizing solution. Carbonyl groups were detected by western blot to evaluate levels of oxidative modification of proteins.

### 2.9. TUNEL Assay

Cells permeabilized with 0.1% TritonX-100 were cultured with TdT-UTP nick end labeling (TUNEL) for 1 h at 37°C. FITC-labeledTUNEL-positive cells were observed using a fluorescence microscope. Red fluorescent cells were considered apoptotic cells.

### 2.10. siRNA Transfection

The siRNA specifically targeting ERK1/2 (Thermo Fisher Scientific) mixed in HiPerFect transfection reagent (Qiagen) at a final concentration of 40 nM was used to transiently transfect cells. The transfected cells were treated with or without various stimuli and then subjected to western blot analysis.

### 2.11. Western Blot Analysis

Extracted protein lysates were separated by SDS-polyacrylamide gels and electrotransferred onto polyvinylidene difluoride membranes. Membranes were blocked with TBST containing 5% nonfat milk. Then, membranes were probed with primary antibodies against p-ERK, p-JNK, p-P38, *β*-tubulin, NLRP3, and IL1*β* (Cell Signaling Technology, Shanghai, China) (1 : 1000) at 4°C. After incubating overnight, membranes were washed and probed with second antibody for another 1 h. Finally, western blot bands were captured and visualized using the ChemiDoc imaging system.

### 2.12. Statistical Analysis

All data were analyzed using SPSS 23.0 software. *T*-test was used to compare the difference between the two groups. Results are expressed as mean ± standard deviation. *P* < 0.05 indicates a statistically significant difference.

## 3. Results

### 3.1. Verbenalin, Hastatoside, and Acteoside Are the Main Active Ingredients of TAEV

**Figure 1:**
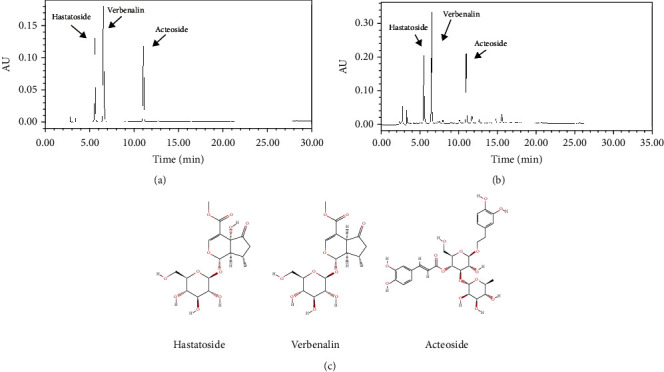
Fingerprint analysis of TAEV by HPLC. (a) Chromatograms of mixed standards. (b) TAEV samples. (c) Molecular structures of verbenalin (C_17_H_24_O_10_, CAS: 548-37-8), hastatoside (C_17_H_24_O_11_, CAS: 50816-24-5), and acteoside (C_29_H_36_O_15_, CAS: 61276-17-3) were gained from PubChem.

Medicine fingerprint analysis by HPLC showed that TAEV was mainly consisted of verbenalin, hastatoside, and acteoside (Figures 1(a)–1(c)). Among them, verbenalin accounts for the largest proportion.

### 3.2. TAEV Attenuates ADR-Induced Renal Injury *In Vivo*

**Figure 2:**
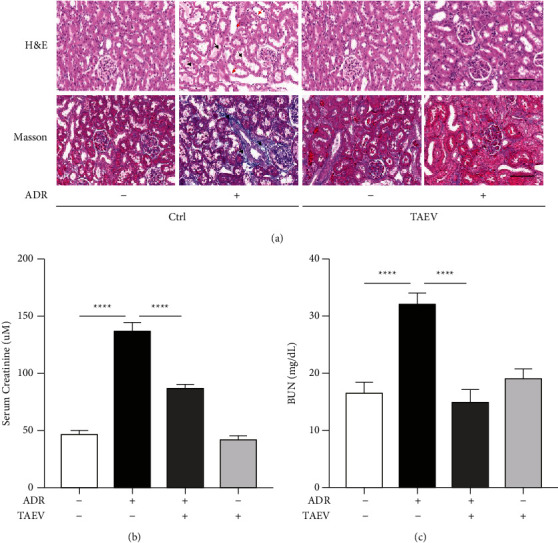
TAEV attenuates ADR-induced renal injury *in vivo*. (a) Renal histological changes were identified using H&E and Masson staining. Bar = 100 *μ*m. In H&E staining, red arrows showed tubular atrophy and black arrows showed tubular necrosis and strong desquamation. In Masson stain, arrows showed increased collagen deposition in the renal tubule interstitium. (b) The changes of renal functions (BUN and Scr). *P* values are indicated at the top of the spots. ^*∗∗∗∗*^*P* < 0.0001.

Verbena is extensively used in the treatment of renal diseases in traditional Chinese medicine. An ANN model was established to examine the effect of verbena on renal injury *in vivo*. According to the histological investigation, DOX significantly produced tubular atrophy, necrosis, and strong desquamation in kidneys, as well as increased collagen deposition, resulting in renal tubulointerstitial fibrosis, whereas TAEV reduced these tubular lesions (Figure 2(a)). In addition, TAEV reduced the ADR-induced worsening of renal function, as evidenced by lower Scr and BUN in the treatment groups (Figure 2(b)). These findings revealed that TAEV minimized ADR-induced renal injury *in vivo*, with a particular protective impact on renal tubules.

### 3.3. TAEV Ameliorates ADR-Induced Renal Tubular Epithelial Cell Injury *In Vitro*

**Figure 3:**
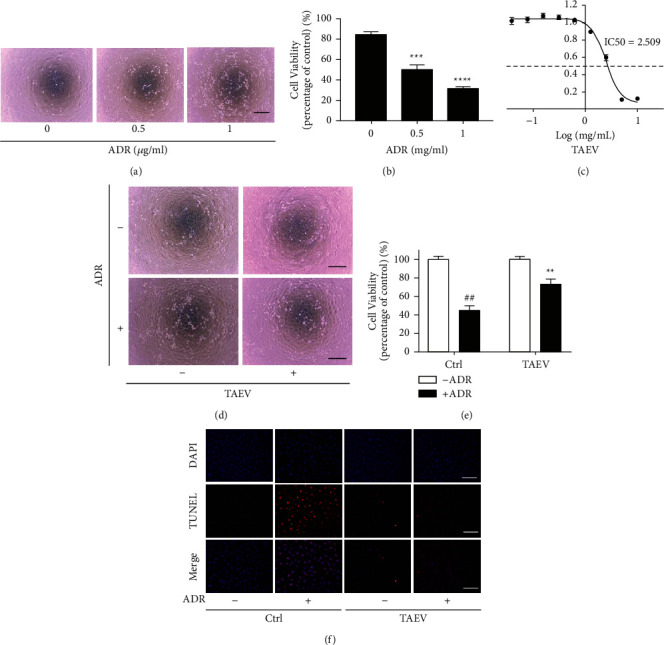
TAEV ameliorates ADR-induced renal tubular epithelial cell injury. (a and b) Cells were insulted with ADR (0, 0.5, 1 *μ*g/mL) for 24 h (bar = 100 *μ*m). Cell viability was measured using CCK-8. (^*∗∗∗*^*P* < 0.001 and ^*∗∗∗∗*^*P* < 0.0001) (c) IC50 of TAEV on NRK-52E cells. IC50 was calculated according to the cell viability of cells treated with different concentrations (0, 0.0390625, 0.078125, 0.15625, 0.3125, 0.625, 1.25, 2.5, 5, and 10 mg/mL) using Graphpad prism software. (d and e) Cells pretreated with TAEV (1 mg/mL) were challenged with ADR (1 *μ*g/mL) for 24 h (bar = 100 *μ*m). Cell viability was tested. (^##^*P* < 0.01 versus control and ^*∗∗*^*P* < 0.01 versus ADR). (f) Cells were treated with ADR (1 *μ*g/mL) and TAEV (1 mg/mL) for 24 h and then stained with TUNEL and DAPI to detect the apoptotic cells.

To further determine the impact of TAEV on renal tubules, an ADR-induced renal tubular epithelial cell injury model was developed *in vitro*. As shown in Figures 3(a) and 3(b), ADR induced a disruption of cells and loss of cellular viability, while TAEV improved the morphological damage changes of cells and rescued the decreased cellular viability (Figures 3(c)–3(e)). In addition, TUNEL staining showed that TAEV mitigated ADR-induced cell apoptosis (Figure 3(f)), suggesting that TAEV might be a novel protective drug against ADR-induced renal tubular epithelial cell injury.

### 3.4. TAEV Attenuates ADR-Induced Oxidative Injury in Tubular Cells

**Figure 4:**
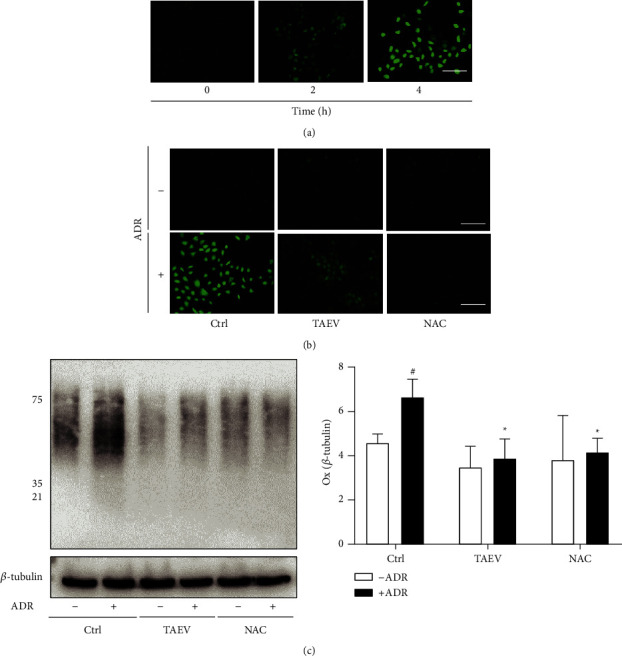
TAEV ameliorates ADR-induced oxidative stress injury in the cells. (a) Cells were insulted with ADR (1 *μ*g/mL) for several times (0, 2, and 4 h and then incubated with DHE agent for another 40 min (bar = 100 *μ*m). (b) Cells pretreated with TAEV (1 mg/mL) and NAC (10 *μ*M) for 1 h were challenged with ADR and then detected using a ROS detection kit. (c) Cells pretreated with TAEV (1 mg/mL) and NAC (10 *μ*M) for 1 h were stimulated with ADR (1 *μ*g/mL) for another 4 h. Then, cellular lysates were analyzed using a OxyBlot protein oxidation detection kit. (^#^*P* < 0.05 versus control and ^*∗*^*P* < 0.05 versus ADR).

Accumulated evidence showed that oxidative stress injury underlies the cytotoxicity of ADR. As shown in Figure 4(a), ADR-induced overproduction of ROS, while TAEV significantly inhibited the changes, similar to the effect of NAC, a classic antioxidant (Figure 4(b)). In addition, TAEV suppressed ADR-induced oxidative modification of proteins as same as the role of NAC (Figure 4(c)), suggesting that TAEV attenuated ADR-induced oxidative stress injury in renal tubular epithelial cells.

### 3.5. TAEV Ameliorates ADR-Induced Renal Tubular Injury by Inhibiting Oxidative Stress-Mediated Phosphorylation of MAPK/ERK1/2 and Activation of NLRP3

**Figure 5:**
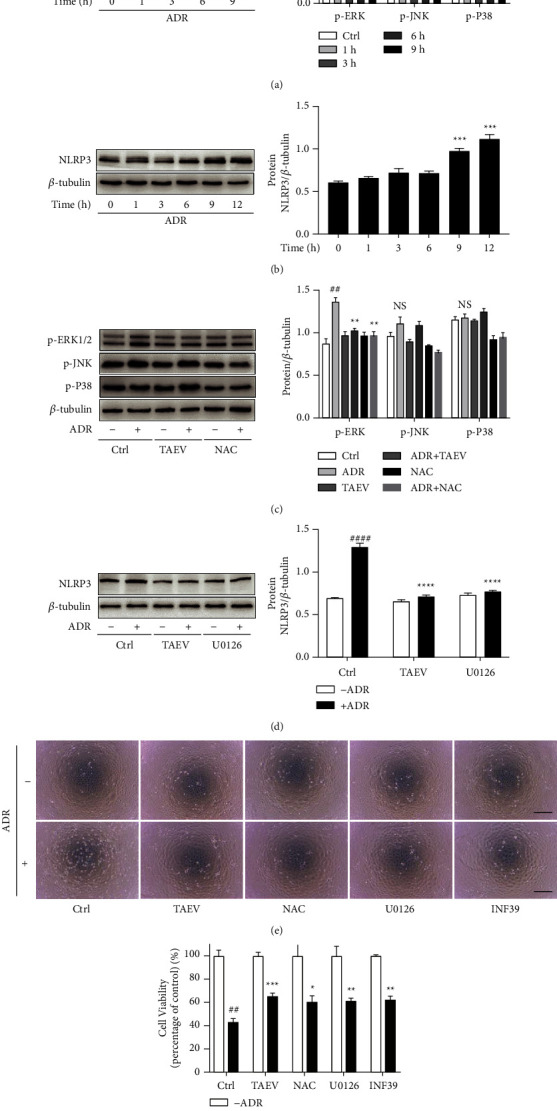
TAEV attenuates ADR-induced renal tubular injury via inhibition of ROS-mediated ERK1/2 and NLRP3 activation. (a and b) Cells were incubated with ADR (1 *μ*g/mL) for corresponding times (^*∗*^*P* < 0.05, ^*∗∗*^*P* < 0.01, and ^*∗∗∗*^*P* < 0.001). (c and d) Cells pretreated with TAEV (1 mg/mL) and NAC (10 *μ*M) for 1 h were incubated with ADR for another 9 h. (^##^*P* < 0.01, ^####^*P* < 0.0001 versus control, ^*∗∗*^*P* < 0.01 and ^*∗∗∗∗*^*P* < 0.0001 versus ADR). (e and f) Cells were pretreated with TAEV (1 mg/mL), NAC (10 *μ*M), U0126 (20 *μ*M), and INF39 (100 *μ*M) for 1 h and incubated with ADR (1 *μ*g/mL) for another 24 h. Cell viability was tested using CCK-8. (^##^*P* < 0.01 versus control and ^*∗*^*P* < 0.05, ^*∗∗*^*P* < 0.01, and ^*∗∗∗*^*P* < 0.001 versus ADR).

Enough evidence revealed that MAPK and NLRP3 signaling pathways were under the trigger of oxidative injury in cells. The western blot analysis showed that ADR induced the phosphorylation of MAPKs (ERK1/2, JNK, P38) and activation of NLRP3 (Figures 5(a) and 5(b)). Interestingly, TAEV inhibited the phosphorylation of ERK1/2 and activation of NLRP3 similar with the role of NAC, but not the phosphorylation of JNK and P38 (Figures 5(c) and 5(d)). Similar to the protective effect of TAEV, the antioxidant NAC as well as inhibitors of ERK (U0126) and NLRP3 (INF39) both ameliorated ADR-induced cell disruption and recovered the loss of cell viability (Figures 5(e) and 5(f)), suggesting that the inhibition of ROS-mediated activation of ERK1/2 and NLRP3 might play a vital role in the improvement of TAEV against ADR-induced renal tubular injury.

### 3.6. Inhibition of the ERK1/2-mediated NLRP3 Signaling Pathway Participates in the Protection of TAEV against ADR-Induced Renal Cell Injury

**Figure 6:**
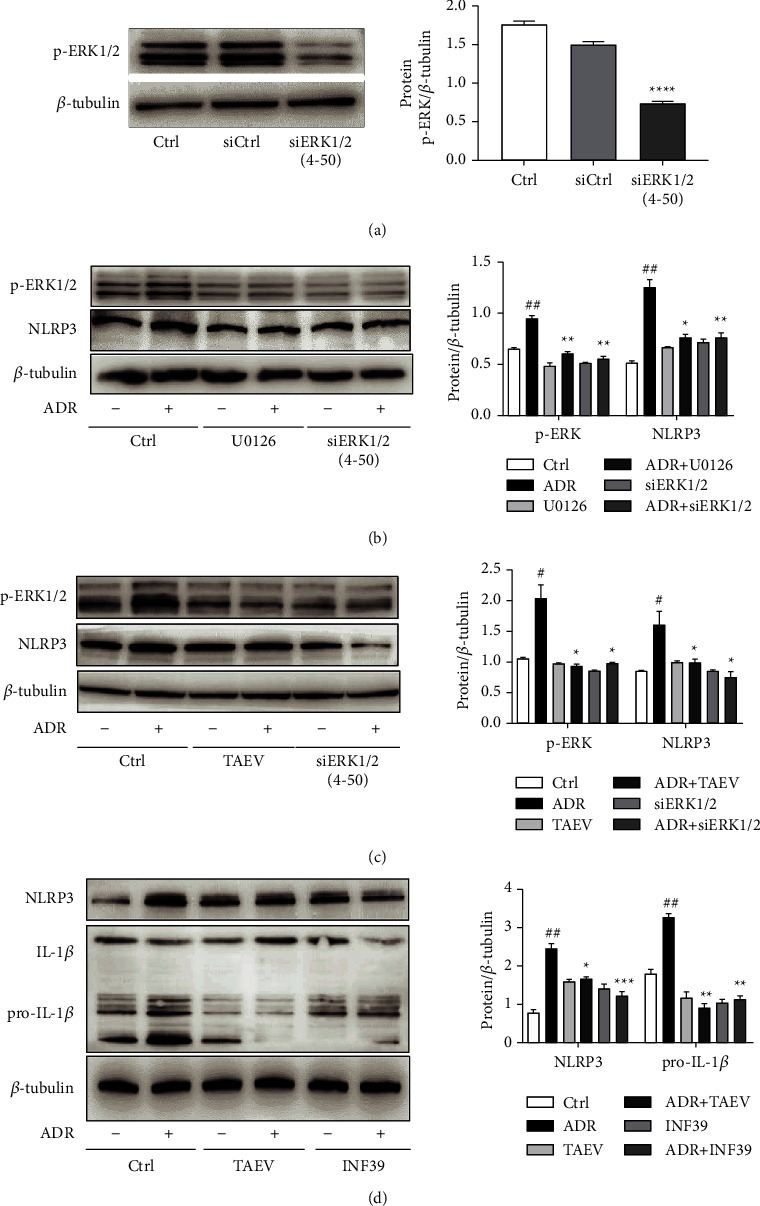
TAEV inhibited ADR-induced ERK1/2-mediated activation of NLRP3 signal pathway. (a) Knocking down ERK1/2 with specific siRNA (^*∗∗∗∗*^*P* < 0.0001). (b) Cells were silenced by ERK1/2 specific siRNA or pretreated with U0126 (20 *μ*M). Then, cells were challenged with ADR (1 *μ*g/mL) for another 9 h (^##^*P* < 0.01 versus control; ^*∗*^*P* < 0.05 and ^*∗∗*^*P* < 0.01 versus ADR). (c) Cells were silenced by ERK1/2 specific siRNA or pretreated with TAEV (1 mg/mL) and then insulted with ADR (1 *μ*g/mL) for another 9 h (^#^*P* < 0.05 versus control and ^*∗*^*P* < 0.05 versus ADR). (d) Cells were pretreated with TAEV (1 mg/mL) and INF39 (100 *μ*M) for 1 h and then stimulated with ADR (1 *μ*g/mL) for another 12 h (^##^*P* < 0.01 versus control and ^*∗*^*P* < 0.05, ^*∗∗*^*P* < 0.01, and ^*∗∗∗*^*P* < 0.001 versus ADR).

To better understand the relationship between ERK1/2 and NLRP3 in ADR-induced tubular cell injury, we observed the changed expression of NLRP3 in injured cells treated with ERK1/2 siRNA (Figure 6(a)). As shown in Figure 6(b), silence of ERK1/2 by siRNA and inhibition of phosphorylated ERK1/2 by specific inhibitor U0126 both suppressed the activation of NLRP3, similar to the role of TAEV (Figure 6(c)). It has been found that activation of NLRP3 contributes to the mature and release of IL1*β* in cells leading to inflammatory response. As shown in Figure 6(d), TAEV inhibited the ADR-induced activation of IL1*β* as same as the role of NLRP3 inhibitor INF39. All outcomes indicated that TAEV could mitigate ADR-induced renal tubular cell injury via ERK1/2-mediated NLRP3 signaling pathway.

## 4. Discussion

In the current study, we confirmed that ROS-mediated phosphorylation of ERK1/2 followed by activation of NLRP3 plays a vital role in ADR-induced renal tubular epithelial cell injury. Our study firstly determined that TAEV attenuates ADR-induced tubular injury via suppressing ROS-ERK1/2-mediated NLRP3 signaling pathway.

Tubular epithelial cells usually bear the brunt of various injuries, leading to the progression of CKD [12]. Therapies to ameliorate tubular epithelial cell injury is the key to delay CKD progression. ADR has significant nephrotoxicity and can be used to mimic renal injury during CKD progression [13]. Our study confirmed that ADR triggers severe renal tubular epithelial cell injury and induces markedly oxidative stress injury in cells. Moreover, we discovered that TAEV, an extract of a natural herb-verbena, significantly improved ADR-induced tubular cell injury.

Verbena is a traditional Chinese natural herb applied in treating various inflammatory diseases such as hepatitis, gonorrhea, dysentery, malaria, nephritis edema, and so on [5–9]. Our clinical practice discovered that verbena significantly delayed the progression of renal function and reduced proteinuria in patients with diabetic nephropathy. In the current study, we determined that the aqueous extract of verbena attenuated ADR-induced damage and oxidative stress injury in tubular epithelial cells. Previous studies reported that verbena is consisted of verbenalin, hastatoside, acteoside, luteolin, ursolic acid, oleanolic acid, and other bioactive compounds [14]. Our study displayed that verbenalin, hastatoside, and acteoside are the main ingredients of verbena. Acteoside has been reported to reduce proteinuria, improve renal function, and slow down renal fibrosis [15]. In addition, verbenalin and hastatoside ameliorate cerebral infarction and prostatitis through antioxidative and anti-inflammatory properties [16, 17], suggesting their potential renal-protective role in kidney diseases. The protective role of TAEV against ADR-induced renal tubular injury might be based on the combined effects of the three ingredients.

Accumulated evidence indicate that oxidative stress injury is intimately associated with the development and incidence of tubular cell injury [18, 19]. Our previous study confirmed that oxidative stress underlines the nephrotoxicity of ADR [20]. Oxidative stress injury is traditionally considered to be induced by the overproduction of ROS due to the imbalance of oxidative and antioxidant systems in the cells. It has been shown that ROS controls endoplasmic reticulum stress, apoptosis, and other signaling pathways that contribute to tubular cell damage in acute kidney injury, indicating that the modulation of signaling pathways plays a crucial role in ROS-mediated renal injury [21]. ROS is the upstream trigger to various damage signaling pathways, among which MAPKs and NLRP3 signaling pathway were reported to play a vital role in ADR-induced renal injuries [11]. MAPKs (including ERK1/2, JNK, and p38) is perceived as an important sensor in the cells in response to external stimuli and plays an important role in cell proliferation, differentiation, transformation, and apoptosis [22]. Activation of NLRP3 inflammasome contributes to the maturation and release of proinflammatory factors such as IL-1*β* and IL-18, which is a vital component of inflammatory response and plays an important role in the progression of kidney diseases [23]. Our study confirmed that ADR significantly induced the phosphorylation of all MAPK pathways and the activation of NLRP3, meanwhile TAEV inhibited the activation of NLRP3 and the phosphorylation of ERK1/2, but not JNK and P38. In addition, both inhibitors of ERK1/2 and NLRP3 could improve ADR-induced renal tubular cell injury, indicating that ERK1/2 and NLRP3 both play a vital role in the protection of TAEV against ADR-induced tubular injury.

NLRP3 and ERK are both the downstream activated targets of ROS. In most cases, MAPK acts as a switch regulating various damage signaling pathways. Herein, we conjectured that NLRP3 might be activated following by the phosphorylation of ERK1/2. Our data showed that the silence or inhibition of ERK1/2 could significantly inhibit the activation of NLRP3, suggesting that ERK1/2 was an important upstream initiator of NLRP3 and TAEV protected renal tubular cells against ADR via suppressing the ERK1/2-mediated activation of NLRP3 signal pathway:

## 5. Conclusions

In summary, our study revealed that ROS-mediated phosphorylation of ERK1/2 and activation of NLRP3 play a vital role in ADR-induced renal tubular injury and TAEV could protect renal tubular cells against ADR toxicity via inhibition of ROS/ERK1/2-mediated NLRP3 signal pathway. Due to this property, TAEV might serve as a promising reno-protective agent in delaying the progression of CKD and ROS/ERK1/2-mediated NLRP3 signal pathway might be a vital target in treating kidney diseases.

## Data Availability

The experimental data used to support the findings of this study are included within the article.
